# Paracrine effects of haematopoietic cells on human mesenchymal stem cells

**DOI:** 10.1038/srep10573

**Published:** 2015-06-01

**Authors:** Shuanhu Zhou

**Affiliations:** 1 Department of Orthopedic Surgery, Brigham and Women’s Hospital and Harvard Medical School, Boston, Massachusetts 02115, USA; 2 Harvard Stem Cell Institute, Harvard University, Cambridge, Massachusetts 02138, USA

## Abstract

Stem cell function decline during ageing can involve both cell intrinsic and extrinsic mechanisms. Bone and blood formation are intertwined in bone marrow, therefore haematopoietic cells and bone cells could be extrinsic factors for each other. In this study, we assessed the paracrine effects of extrinsic factors from haematopoietic cells on human mesenchymal stem cells (MSCs). Our data showed that haematopoietic cells stimulate proliferation, osteoblast differentiation and inhibit senescence of MSCs; TNF-α, PDGF-β, Wnt1, 4, 6, 7a and 10a, sFRP-3 and sFRP-5 are dominantly expressed in haematopoietic cells; the age-related increase of TNF-α in haematopoietic cells may perform as a negative factor in the interactions of haematopoietic cells on MSCs *via* TNF-α receptors and then activating NF-κB signaling or Wnt/β-catenin signaling to induce senescence and reduce osteoblast differentiation in MSCs. In conclusion, our data demonstrated that there are paracrine interactions of haematopoietic cells on human MSCs; immunosenescence may be one of the extrinsic mechanisms by which skeletal stem cell function decline during human skeletal ageing.

Mammalian stem cells are maintained and regulated by their local tissue microenvironment, the niche^1^. Haematopoietic stem cell niche is well documented that provides a model for understanding stem cell niches^1^. Mesenchymal stem cells or marrow stromal cells (MSCs) have been demonstrated to be precursors of several different cellular lineages, including bone-forming osteoblasts. MSCs function as key regulators and niche factors of haematopoietic stem cells (HSCs) in bone marrow^1^,^2^,^3^. Bianco^4^ hypothesized a dual sinusoidal niche of MSCs and HSCs in bone marrow in which two kinds of stem cells share an identical microanatomical location in the bone/bone marrow organ. However, the interactions of haematopoietic cells on human MSCs (hMSCs) are not fully understood.

Bone marrow is soft blood-forming tissue that fills the cavities of bones and contains fat, bone cells, stromal cells, immature and mature blood cells, and is important for the proper development of the immune system^5^,^6^,^7^,^8^,^9^. Within bone marrow, as well as outside of it, cytokines produced by immune cells have important effects on regulating bone homeostasis^6^,^7^,^8^,^9^. Osteoimmunology is defined as the research area focusing on the crosstalk between the immune system and the skeletal system^6^,^7^,^8^,^9^. Emerging clinical and molecular evidences demonstrate that senile osteoporosis is an immune-mediated disease^8^,^9^. Animal studies demonstrated that haematopoietic cells, such as HSCs^10^, T-cells^7^,^11^ and megakaryocytes^12^, have reciprocal regulatory interactions on bone cells. Studies have shown that MSCs have unique immunoregulatory properties and there are bidirectional interactions between MSCs and immune system, which determine the outcome of MSC-mediated tissue repair processes^13^,^14^. Tumor necrosis factor α (TNF-α) is a multifunctional cytokine that is produced by a variety of immune cells including T cells, B cells, NK cells and macrophages^15^,^16^. TNF-α has a central role in bone pathophysiology and its action in the skeleton results in increased bone resorption by stimulation of osteoclastogenesis and impaired bone formation by suppressing recruitment of osteoblasts from progenitor cells, inhibiting the expression of matrix protein genes, and stimulating expression of genes that amplify osteoclastogenesis^17^. Modulation of TNF-α restored regenerative osteoblastogenesis in aged mice^18^.

Several lines of evidence indicate that the decline in stem cell function during ageing can involve both cell intrinsic and extrinsic mechanisms^19^. The bone and blood formation are intertwined in bone marrow^5^, therefore, haematopoietic cells and bone cells could be extrinsic factors for each other in bone marrow environment. There is growing evidence in animal studies^20^ and invertebrate model^21^ that the stem cell niche, one of the extrinsic mechanisms, is important for the regulation of cellular ageing in stem cells. We^22^,^23^,^24^ uncovered that there are age-related intrinsic changes in hMSCs. In this study, by using an *in vitro* transwell co-culture platform (Fig. 1a and Supplementary Fig. 1), we assess the paracrine interactions of human bone marrow haematopoietic cells on mesenchymal stem cells. Our data demonstrate that there are paracrine effects of human bone marrow haematopoietic cells *via* soluble factors, such as TNF-α, PDGF-β or Wnts etc., on hMSCs that may be one of the extrinsic mechanisms of skeletal stem cell function decline during human skeletal ageing.

## Results

### Effects of human bone marrow haematopoietic cells on proliferation and senescence of MSCs

**Figure 1:**
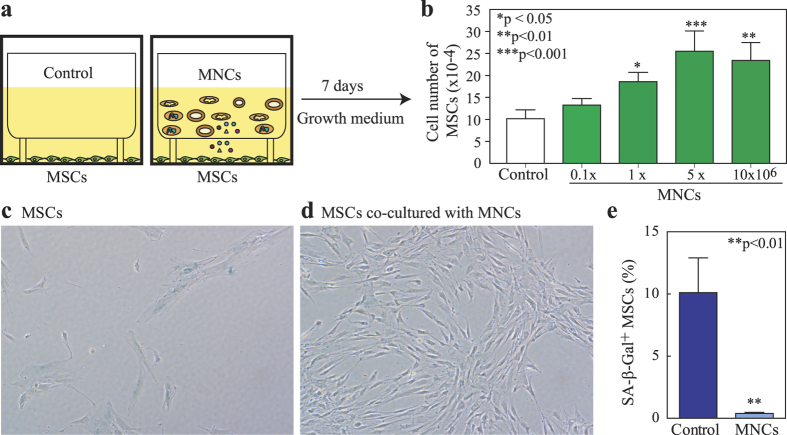
Human bone marrow haematopoietic cells stimulate proliferation and diminish senescence of human MSCs. (**a**) The co-culture system of MSCs ± MNCs. (**b**) MNCs dose-dependently stimulate cell proliferation in MSCs (inserts of MNCs *vs*. empty insert controls, n = 3, one way ANOVA with Tukey post-hoc test). (**c**) Photomicrograph shows MSCs in empty control insert stained with SA-β-Gal (200x). (**d**) Photomicrograph shows MSCs co-cultured with MNCs stained with SA-β-Gal (200x). (**e**) Quantitative data of SA-β-Gal^+^ cells in MSCs (n = 6, Mann-Whitney test).

The vast majority (93-98%) of low-density human bone marrow mononuclear cells (MNCs) are Lin^+^ haematopoietic cells based on our assays of human MNCs with magnetic activated cell sorting (Miltenyi Biotec). To test the effects of haematopoietic cells on proliferation of human MSCs, we used a co-culture system (Fig. 1a) that MNCs were placed in cell culture inserts, and MSCs were cultured on the bottom of the dishes. The 0.4 μm pore size of cell culture insert (Nunc Inserts, Thermo Scientific) allows proteins or small molecules to transport through the polycarbonate membrane, but not cells. Human MSCs (obtained from a 78-year-old female subject, 78 F) were seeded in 6-well plates at 1 × 10^4^ cells per well with empty transwell controls or co-cultured with 0.1-10 × 10^6^ of human MNCs (62F) per transwell insert (Fig. 1a,b). After 7 days cultivation in MSC growth medium (MEM-α with 10% FBS-HI, 100 U/mL penicillin and 100 μg/mL streptomycin), cell number was determined by hemacytometer. MNCs (≥1 × 10^6^) significantly stimulated MSC proliferation (Fig. 1b). The stimulation on proliferation of MSCs by haematopoietic cells was confirmed by MSCs (76F) ± MNCs (60 F), MSCs (78F) ± MNCs (46 M), and MSCs (90 F) ± MNCs (61 F) (Supplementary Fig. 2a).

To determine whether the haematopoietic cellular microenvironment has inhibitory effects on the ageing of hMSCs, or even rejuvenates aged hMSCs. Human MSCs (64 M) were cultured in 6-well plates at 1 × 10^4^ cells per well in MEM-α with 10% FBS-HI. MNCs (62 F) were put into transwell insert; empty inserts were used as controls. One week after co-culture, MSCs were stained for senescence associated β-galactosidase (SA-β-Gal). MNCs significantly decreased the percentage of SA-β-Gal^+^ cells (blue) in MSCs. Human MSCs control (Fig. 1c) had fewer cells and many of them had an enlarged, flattened morphology, while the MSCs with MNCs co-culture contained more cells with a spindle or cuboid shape (Fig. 1d). Human MNCs (5 × 10^6^ cells per insert) co-cultures significantly decreased SA-β-Gal^+^ cells (p < 0.01) (Fig. 1e), implying that haematopoietic cells may rejuvenate human MSCs of the elderly.

### Effects of human bone marrow haematopoietic cells on osteoblastogenesis of MSCs

**Figure 2:**
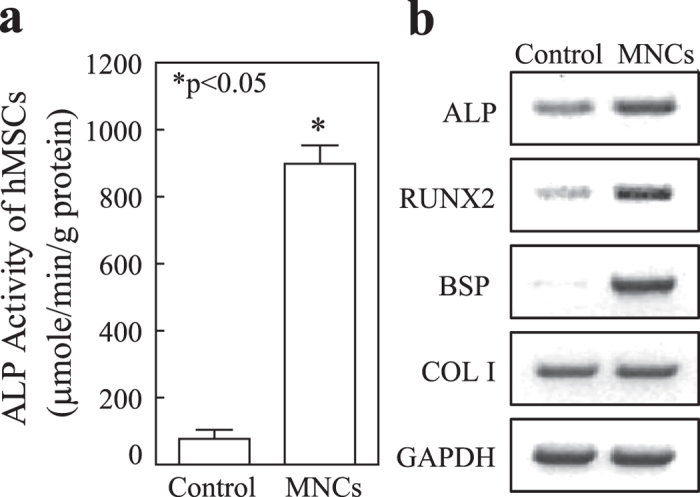
Haematopoietic cells stimulate osteoblast differentiation of human MSCs. (**a**) ALP activity of MSCs ± MNCs, MNCs inserts (n = 3) *vs*. empty insert controls (n = 6) (Mann-Whitney test). (**b**) Agarose gel electrophotogram shows RT-PCR products for osteoblast genes, ALP, RUNX2, BSP and COL I in MSCs co-cultured with empty control or MNCs inserts.

Human MSCs (90 F) were cultured in 6-well-plates until confluence. The medium was changed to MEM-α with 1% FBS-HI plus osteogenic supplements as we described^22^ and cell culture inserts were put into the wells with or without 5 × 10^6^ MNCs (61F). At day 7, MNCs inserts stimulated alkaline phosphatase (ALP) activity in MSCs, 11.7-fold compared with control (p < 0.05, Mann-Whitney test) (Fig. 2a). Expression of osteogenic genes, such as *ALP*, Runt-Related Transcription Factor 2 (*RUNX2*), Bone Sialoprotein (*BSP*) but not Collagen type I (*COL I*), were also stimulated by MNCs inserts (Fig. 2b). In contrast to MNCs, mouse embryonic fibroblasts (MEFs) insert (1 × 10^6^/insert, n = 3) significantly inhibited the ALP activity of human MSCs (MEFs inserts *vs.* empty insert controls, p < 0.05) (Supplementary Fig. 3b). These data indicate that soluble factors secreted from MNCs, but not the culture environment changes, have positive effects on osteoblastogenesis in MSCs.

The stimulation on ALP by MNCs was confirmed in MSCs (76 M) co-cultured with MNCs obtained from a 48-year-old female subjects (Supplementary Fig. 3a). Interestingly, compared with MNCs obtained from the young subject, MNCs obtained from a 67-year-old subject had no significant stimulation on ALP activity in MSCs.

### The convergences and divergences in gene profile of human MNCs and MSCs

**Figure 3:**
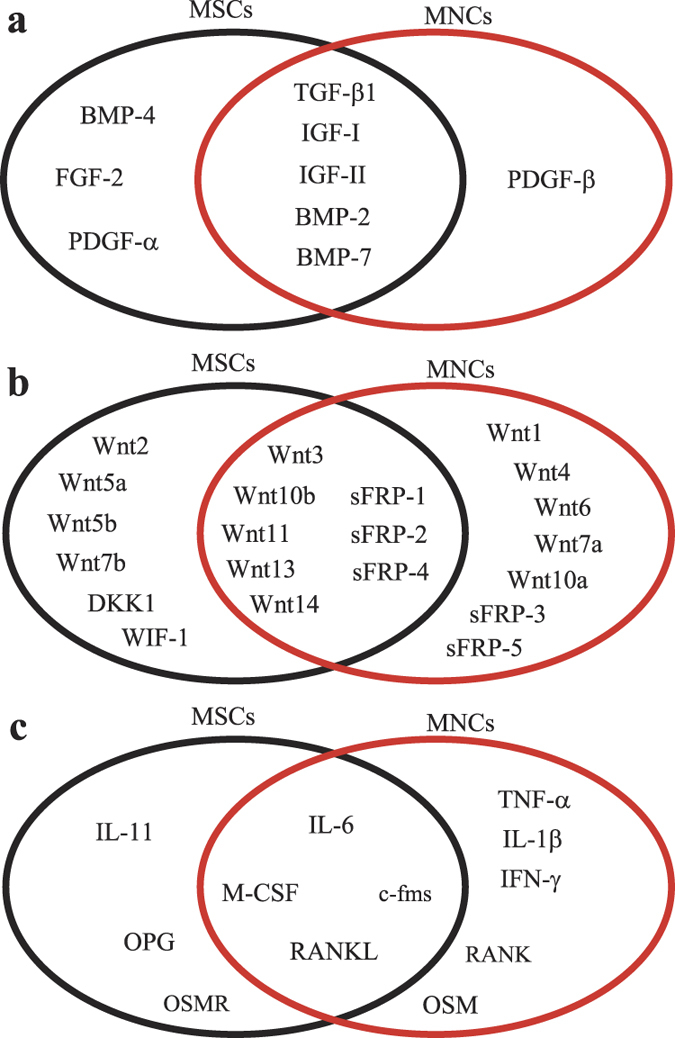
Venn analyses of the convergences and divergences in gene profile of human MNCs and MSCs. (**a**) Growth factors, (**b**) Wnt-related factors, (**c**) cytokines and factors that is important in the interactions between haematopoietic lineage osteoclasts and osteoblasts or MSCs.

In order to determine the factors secreted from human MNCs that may response for the effects of MNCs on MSCs, we performed RT-PCR (Supplementary Fig. 4) to evaluate the gene profile in human MSCs and MNCs obtained from two subjects, a young male (17 M) and an old male (64 M) subjects. Our data as summarized by Venn analysis (Fig. 3) reveals the convergences and divergences in gene expression of growth factors (Fig. 3a & Supplementary Fig. 4a), Wnt-related factors (Fig. 3b & Supplementary Fig. 4b), cytokines (Fig. 3c & Supplementary Fig. 4c) and factors that are important to the interactions between osteoclasts and osteoblasts/MSCs (Fig. 3c and Supplementary Fig. 4d&e) in human MNCs and MSCs.

We first determined the gene profile of growth factors that are well known for their influence on either proliferation or differentiation of human MSCs. Our data showed that PDGF-β is dominantly expressed in human MNCs; BMP-4, FGF-2 and PDGF-α are dominantly expressed in human MSCs; TGF-β1, IGF-I, IGF-II, BMP-2 and BMP-7 are expressed in both MNCs and MSCs (Fig. 3a & Supplementary Fig. 4a). Secondly, we analyzed the gene profile of canonical Wnts (Supplementary Fig. 4b1), non-canonical Wnts (Supplementary Fig. 4b3) and Wnt inhibitors (Supplementary Fig. 4b4) in MSCs and MNCs. Our data showed that Wnt1, 4, 6, 7a, and 10a are dominantly expressed only in MNCs, but not MSCs; Wnt 2, 7b, 5a, and 5b are mainly expressed in hMSCs; other Wnts, such as Wnt 3, 10b, 11, 13, and 14, are expressed in both (Fig. 3b), suggested that Wnt1, 4, 6, 7a and 10b, which are mainly expressed in MNCs, might have roles in the haematopoietic cellular environmental interactions with MSCs. Human MNCs dominantly express Wnt inhibitors, secreted Frizzled-related peptide (sFRP) -3 and -5 (Supplementary Fig. 4b4), suggesting that there are roles of sFRP-3 and sFRP-5 in the interactions of haematopoietic cells on hMSC function and ageing.

We then assessed the cytokine gene profile in both human MNCs and MSCs. Our data showed (Fig. 3c and Supplementary Fig. 4c) that TNF-α, IL-1β and IFN-γ are only expressed in human MNCs; IL-11 and dominantly expressed in human MSCs; IL-6 is expressed in both MNCs and MSCs. Macrophage-colony stimulating factor (M-CSF), receptor activator of NF-κB ligand (RANKL), and osteoprotegerin (OPG) and their receptors, c-fms and RANK are important in the interactions between haematopoietic lineage osteoclasts and osteoblasts/MSCs^25^. Our data showed (Fig. 3c & Supplementary Fig. 4d) that RANK is only expressed in human MNCs and OPG is only expressed in human MSCs; RANKL, M-CSF and its receptor c-fms are expressed in both. Oncostatin M (OSM) and its receptor (OSMR) are coupling factors between monocytes/macrophages and MSCs^26^. Our data showed (Supplementary Fig. 4e) that human MNCs expresses OSM and its receptor OSMR is only expressed in MSCs, suggested that OSM/OSMR signaling may be one of the mechanisms that induce osteoblast differentiation in MSCs by haematopoietic cells in human bone marrow.

### Effects of age on TNF-α signaling in human MNCs and MSCs

**Figure 4:**
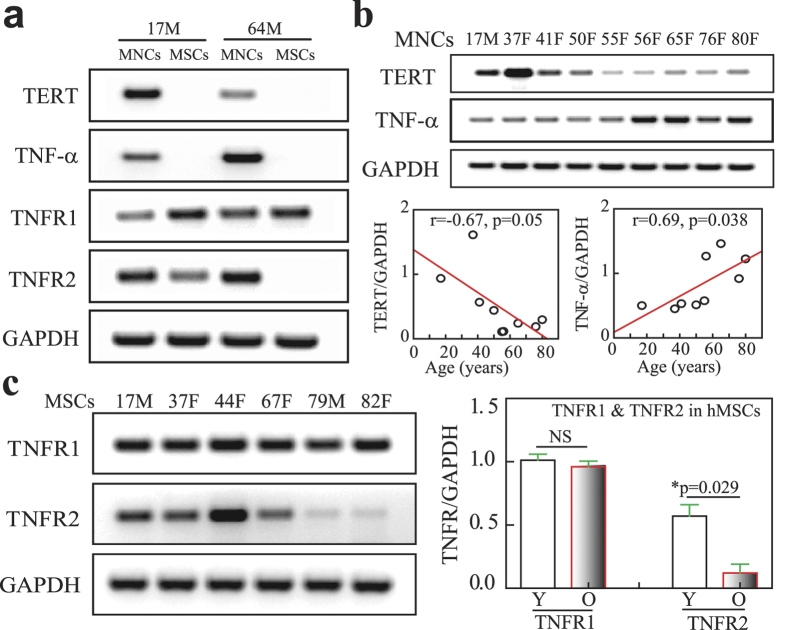
The effects of age on TNF-α signaling in human MNCs and MSCs. (**a**) The expression of TNF-α and its receptors as well as TERT in MNCs and MSCs obtained from two subjects. (**b**) The age-related changes of TERT and TNF-α gene expression in MNCs (n = 9, Pearson correlation). (**c**) The age-related changes of TNF-α receptor 1 (TNFR1) and 2 (TNFR2) in MSCs (NS, not significant; *p = 0.029, Mann-Whitney test).

Human MSCs (passage 2) obtained from 17-year-old male (17 M) and 64-year-old male (64 M) subjects were taken as examples of MSCs to compared the gene expression for TNF-α pathway with their MNCs as shown by RT-PCR (Fig. 4a). There was TNF-α gene expression in MNCs of both subjects, and no detectable TNF-α in MSCs. MSCs express TNFR1 and TNFR2, but MSCs from old (64 M) subject had much lower TNFR2 than MSCs from young (17 M) subject. Human Telomerase Reverse Transcriptase (hTERT) (the catalytic subunit of telomerase) gene was expressed in MNCs, but not in MSCs (Fig. 4a). There were age-dependent decline on hTERT (r = −0.67, p = 0.05) and increase on TNF-α (r = 0.69, r = 0.038, n = 9, Pearson) gene expression (RT-PCR) in human MNCs obtained from 4 young subjects (≤50-year-old subjects) and 5 old subjects (≥55-year-old subjects) (Fig. 4b). Compared with old subjects, TERT gene expression was significantly lower (p < 0.05) and TNF-α was significantly higher (p < 0.05, Mann-Whitney test) in MNCs obtained from young subjects. Additional quantitative RT-PCR data showed that there was no significant (NS) different in TNFR1 (p = 0.89) but a significant decline of TNFR2 (p = 0.029, Mann-Whitney test) gene expression in MSCs from 3 young subjects (17-44-year-old) and 4 old subjects (64-82-year-old) (Fig. 4a,c). These data suggest that there is an age-related change in TNF-α signaling in MSCs and haematopoietic cells of human bone marrow.

### Effects of TNF-α on senescence, cell death, proliferation and osteoblast differentiation of human MSCs

**Figure 5:**
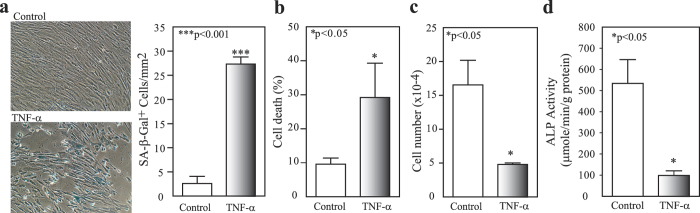
The effects of TNF-α on human MSCs. (**a**) The effects of TNF-α on SA-β-Gal^+^ cells, TNF-α (20 ng/mL, n = 3) *vs*. control (BSA, n = 3); (**b**) cell death, TNF-α (20 ng/mL, n = 6) *vs*. control (n = 3), and (**c**) proliferation, TNF-α treatment (10 ng/mL, n = 3) and control (n = 3), in MSCs (17 M). (**d**) TNF-α significantly inhibited ALP activity of MSCs (17 M) (20 ng/mL of TNF-α treatment, n = 3, *vs*. control, n = 3) (t-test).

Human MSCs (passage 2) obtained from 17-year-old male (17 M) subject were cultured in MEM-α with 10% FBS-HI and antibiotics, and treated with vehicle control (0.1% bovine serum albumin in PBS) or TNF-α (R&D, MN). TNF-α (20 ng/mL, 5 days treatment) induces MSC senescence (10-fold increase *vs*. control) as shown the Senescence-Associated β-Gal positive cells (blue) (Fig. 5a). Cell death were assayed in human MSCs that were treated with 20 ng/mL TNF-α for 10 days without medium changes (trypan blue exclusion assay); there was 3-fold more cell death in treatment groups than the vehicle controls (Fig. 5b). Population kinetics was evaluated with short-term cultures of MSCs. After 7 days treatment, TNF-α (10 ng/mL, twice a week) significantly inhibited the proliferation of MSCs (4.80 ± 0.23 × 10^4^ per 35 mm cell culture dish) compared with vehicle controls (16.53 ± 3.65 × 10^4^) (p = 0.031, Mann-Whitney test) (Fig. 5c). Similar results on the effects of TNF-α on senescence, cell death and proliferation were also observed in MSCs obtained from a 76-year-old male subject (Supplementary Fig. 5a). The inhibitory effect of TNF-α on proliferation of MSCs were also observed in MSCs from 64-year-old male, 70-year-old female and 72-year-old female subjects (Data not shown) and the induction of cell death by TNF-α was also observed in MSCs from 90-year-old female subject (Data not shown). Our results showed that TNF-α (20 ng/mL, twice a week in MEM-α with 1% FBS-HI plus osteogenic supplements as we previous described^22^,^27^) significantly inhibited osteoblast differentiation in MSCs obtained from a 17-year-old male subject (Fig. 5d). Our ALP data were also confirmed that TNF-α significantly inhibited osteoblastogenesis in a dose-dependent manner as indicated the inhibitory on alkaline phosphatase (ALP) activity in MSCs obtained from a 47-year-old female subject (Supplementary Fig. 5b, ANOVA).

### Effects of TNF-α on NF-κB signaling and β-catenin in human MSCs

**Figure 6:**
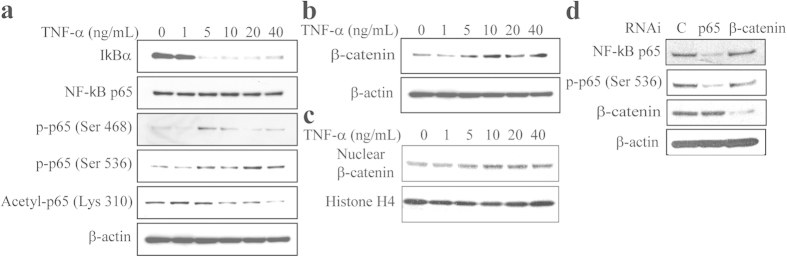
The effects of TNF-α on NF-κB and β-catenin in human MSCs. (**a**) TNF-α treatment for 30 mins activated NF-κB signaling as shown with anti-IκBα, p65, phosphorylated p65, acetylated p65 antibodies by Western blot in human marrow stromal KM101 cells. (**b**) TNF-α stabilized β-catenin proteins in KM101 cells. (**c**) TNF-α increased nuclear β-catenin accumulation in KM101 cells. (**d**) Knockdown p65 or β-catenin with RNAi did not influence the protein levels of each other in KM101 cells.

Our data showed that there is an age-related change in TNF-α signaling in MSCs (lower TNFR2 with age) and in haematopoietic cells (age-related increase of TNF-α gene expression) (Fig. 4), and TNF-α induces senescence, cell death and inhibits proliferation and osteoblast differentiation of MSCs (Fig. 5). To further analysis of TNF-α signaling in human MSCs, we assessed the effects of TNF-α (0-40 ng/mL) on the regulation of intracellular signaling molecules in a human marrow stromal cell line, KM101 cells (Fig. 6a). Our data showed that TNF-α (exceeding 5 ng/mL) induces degradation of IκBα, and then activates NF-κB, as indicated that TNF-α treatment for 30 mins increases phosphorylation of NF-κB p65 subunit at Serine 468 and 536 (Fig. 6a). Acetylation, like phosphorylation, is important for regulating the nuclear function of NF-κB and acetylation of p65 at Lysine 310 is required for full transcriptional activity of p65^28^. Our Western blot data showed that TNF-α increased acetylated p65 Lys 310 at low doses (less than 5 ng/mL) and decreased the acetylation of NF-κB at p65 Lys 310 at high doses (exceeding 10 ng/mL), implied a complex role of TNF-α in human MSCs.

We assessed the effects of TNF-α on Wnt signaling by analyzed its regulation of β-catenin protein levels in human marrow stromal KM101 cells. Our result showed that TNF-α dose-dependently increases β-catenin total proteins (Fig. 6b) and its nuclear accumulation (Fig. 6c). We next assessed whether there are interactions between NF-κB and β-catenin pathways (Fig. 6d). Knockdown of p65 by RNAi (RelA/p65 siRNA, Stealth RNAi duplex siRNA, Invitrogen) reduced total p65 and phosphorylation of NF-κB p65 (Serine 536) levels as shown by Western blotting (Fig. 6d), but that did not influence β-catenin levels. To assess whether β-catenin influences NF-κB p65, we transfected β-catenin siRNA (Stealth RNAi duplex siRNA, Invitrogen) into human marrow stromal KM101 cells. Western blot confirmed that β-catenin proteins were knockdown by β-catenin siRNA (Fig. 6d), but blocking β-catenin did not affect the protein levels of total and phosphorylation of NF-κB p65 (Fig. 6d). Our data implied that NF-κB and β-catenin are two separate pathways in human MSCs.

### Effects of Wnt/β-catenin signaling on senescence and osteoblast differentiation of human MSCs

**Figure 7:**
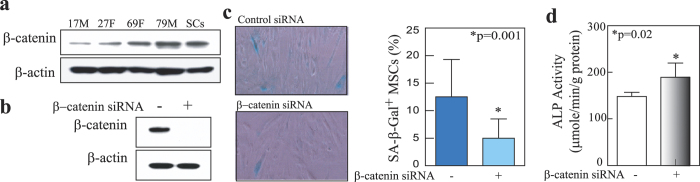
Wnt/β-catenin signaling and senescence of human MSCs. (**a**) An age-related increase of β-catenin protein levels in MSCs. (**b**) Knockdown of β-catenin protein in MSCs was achieved with β-catenin-siRNA (+), but not with non-silencing control siRNA (−). (**c**) Knockdown β-catenin proteins with β-catenin siRNA (n = 12) decreased SA-β-Gal^+^ cells (%) in MSCs, compared with control siRNA (n = 11) (*p = 0.001, Mann-Whitney test). (**d**) Knockdown β-catenin proteins with β-catenin siRNA (n = 6) increased ALP activity in MSCs, compared with control siRNA (n = 7) (*p = 0.02, two-tail unpaired t test with Welch correction).

Our previous studies^29^,^30^ and this study (Fig. 3) showed that human MSCs express many Wnt signaling components, including Wnts and their receptors LRP5/6 and Frizzled. In this study, our Western blotting results showed that the constitutive levels of β-catenin protein levels are higher in MSCs obtained from older subjects (a 69-year-old female, 69 F, and a 79-year-old male, 79 M) and *in vitro* senescent cells (SCs) (hMSCs, 42 F, were cultivated until senescence *in vitro*) (Fig. 7a). This result was confirmed by another cohort of MSCs (Supplementary Fig. 6a), which also expressed higher levels of p53 and p21 mRNA^22^ and proteins (Supplementary Fig. 6a) in aged cells. To assess the role of β-catenin in the senescence of MSCs, we transfected β-catenin siRNA (Stealth RNAi duplex siRNA, Invitrogen) into MSCs (54 M) at 100 pmole per 1 × 10^6^ cells. Western blot confirmed that β-catenin proteins were knockdown by β-catenin siRNA (Fig. 7b). Blocking β-catenin significantly decreased SA-β-Gal positive cells in MSCs (54 M) (Fig. 7c) and increased ALP activity in MSCs (54 M) (Fig. 7d). These data imply that activating Wnt/β-catenin signaling might induce human MSC senescence and reduce osteoblast differentiation.

## Discussion

Bone and blood formation are intertwined in bone marrow^5^. Bianco^4^ hypothesized a dual sinusoidal niche of MSCs and HSCs in bone marrow in which two kinds of stem cells share an identical microanatomical location. Skeletal stem cells/MSCs have been identified *in vivo* in mouse bone marrow by single-cell and lineage tracing analyses^31^,^32^ or *in vivo* transplantation experiments of human bone marrow into nude mice^33^. These recent data demonstrated that the complex interplay of osteogenesis and haematopoiesis in development, physiology, and disease may be seen as rooted into a unique functional interplay of two systems of progenitor/stem cells that takes place in the bone marrow environment at specific sites^33^; soluble factors, such as BMP2 and VEGF^31^, or BMP antagonist^32^, define mouse skeletal stem cells (mSSCs) in bone marrow by regulating the mSSC niche to specify its differentiation toward bone or cartilage. Therefore, haematopoietic cells and bone cells could be niche for each other *in vivo via* soluble factors as shown in Supplementary Fig. 1a. Data suggests that stem cell ageing is controlled at least in part by blood-borne mediators, which change with age and can be manipulated to reverse age-associated dysfunction^20^,^34^,^35^,^36^,^37^. In this study, by using an *in vitro* model of co-cultured haemopoicitc cells and human mesenchymal stem cells (Supplementary Fig. 1b) to study the human blood-borne mediators that may control MSC ageing and to mimic the *in vivo* human MSC niche (Supplementary Fig. 1a). Our data showed that compared with empty insert controls, the inserts of haemopoicitc cells stimulate proliferation (Fig. 1) and osteoblast differentiation (Fig. 2), and inhibit senescence (Fig. 1) in human MSCs, demonstrated that there are positive paracine effects of haemopoicitc cells *via* cell-secreted soluble factors on MSCs. In order to determine the factors secreted from human haemopoicitc cells, we performed RT-PCR to evaluate the gene profile in human MSCs and MNCs (Supplementary Fig. 4). Our data (as summarized in Fig. 3) reveals the convergences and divergences in gene expression of growth factors, Wnt-related factors, cytokines and factors, which are important in the interactions between osteoclasts and osteoblasts/MSCs, in human MNCs and MSCs. Among these growth factors and cytokines, PDGF-β, Wnt1, 4, 6, 7a, and 10a, and OSM are particularly interested because they are dominantly expressed in human MNCs, therefore, these growth factors may response for the positive paracrine effects of haemopoicitc cells on hMSCs.

Platelet-derived growth factors (PDGFs) have important functions in the development of connective tissues^38^. PDGF and other signaling molecules together with MSCs play important roles in bone fabrication processes in which PDGF-BB could function at sites of injury to mobilize the pericytes/MSCs from their abluminal location, stimulate mitotic expansion of these cells and contribute to MSC entering the osteogenic lineage as it exposed to various osteogenic factors such as BMPs and Wnts^39^. Our data showed that PDGF-β was dominantly expressed in haemopoicitc cells and PDGF-α was dominantly in MSCs suggesting their different roles in bone marrow microenvironment and PDGF-β may be one of the paracrine factors that response for the stimulation of proliferation (Supplementary Fig. 2b) and osteoblast differentiation in MSCs. Wnt1 mutations result early-onset osteoporosis and osteogenesis imperfecta in humans and reduce mineralization of MC3T3 cells^40^. Wnt-4 enhanced *in vitro* osteogenic differentiation of MSCs isolated from human adult craniofacial tissues and promoted bone formation *in vivo*^41^. Transgenic mice expressing Wnt4 from osteoblasts were significantly protected from bone loss and chronic inflammation induced by ovariectomy, tumor necrosis factor or natural ageing^42^. Wnt 6 and 10a stimulate osteoblastogenesis^43^. Our previous studies demonstrated that growth factors such as BMPs, TGF-β1 and IGFs are important in osteoblastogenesis and/or chondrocytogenesis of MSCs^29^,^44^,^45^,^46^. Our this study showed that Wnt1, 4, 6, 7a, and 10a were dominantly expressed in haemopoicitc cells and growth factors such as BMPs, TGF-β1 and IGFs were expressed in both haemopoicitc cells and MSCs. The reported data of gene profile, protein array and proteomic analysis of secretome, together with the data of this study showed that there are many secreted factors, such as BMPs, CCLs, CXCLs, FGFs, GDFs, IGFs, ILs, TGFs, VEGF, Wnts etc. from both haemopoicitc cells and MSCs that may involve their paracrine interactions (summarized in supplementary Figure 7).

Immunosenescence is a complex process that negatively impacts the development and function of the immune system, from defects in the haematopoietic bone marrow to defects in peripheral lymphocyte migration, maturation and function^47^, and is an important contributing factor to the development of senile osteoporosis^48^. Our data (Fig. 4b) showed that there is an age-related decline on hTERT gene expression in MNCs, suggested the ageing in MNCs. Ageing is associated with increased levels of circulating cytokines and proinflammatory markers. High level of tumor necrosis factor-α (TNF-α) is associated in the older subject with increased risk of morbidity and mortality^49^. Modulation of TNF-α restored regenerative osteoblastogenesis in aged mice^18^. In order to determine the negative factors secreted by haemopoicitc cells that may induce MSC ageing, we focused on TNF-α because it is only detectable in human haemopoicitc cells (MNCs), but not in human MSCs. Cellular response to TNF-α is mediated through interaction with receptors TNFR1 and TNFR2 and results in activation of pathways that favor both cell survival and apoptosis depending on the cell type and biological context. TNFR1 is expressed by all human tissues and is the major signaling receptor for TNF-α, while TNFR2 is mostly expressed in immune cells and a few of other cells types, and mediates limited biological responses^50^. Our data showed that there is an age-related change in TNF-α signaling in human MSCs (lower TNFR2 with ageing) and their niche, the haematopoietic lineage cells (higher TNF-α expression with ageing) (Fig. 4), and *in vitro*, TNF-α induces senescence, cell death and inhibits proliferation of human MSCs (Fig. 5). TNF-α has a central role in bone pathophysiology and is necessary for stimulation of osteoclastogenesis^17^. It was inconsistency in the literature about the effects of TNF-α on osteoblast differentiation of human MSCs, either stimulation^51^ or inhibitory MSCs^52^,^53^. Our data showed that TNF-α inhibits osteoblast differentiation (Fig. 5 and Supplementary Fig. 5), implied that elevated production of TNF-α by their niche, haemopoicitc cells, in the elderly may be associated with the decline potential of osteogenesis in MSCs.

One of the most important downstream signaling targets activated by TNF-α is the NF-κB transcription factor through various signaling molecules, including TRAF2, RIP, MAP3K, and the IKK complex^54^. These regulatory modifications, which include phosphorylation, ubiquitination, acetylation, sumoylation and nitrosylation, can vary, depending on the nature of the NF-κB-inducing stimulus^55^. There were significantly elevated constitutive levels of NF-κB subunits, p65 and p50, in MSCs derived from adipose tissue from older donors^56^. Our data showed that TNF-α induces degradation of IκBα, and then activates NF-κB, as indicated that TNF-α increase phosphorylation p65 subunit of NF-κB at Serine 468 and 536 (Fig. 6a). Acetylation, like phosphorylation, is important for regulating the nuclear function of NF-κB and acetylation of p65 at Lysine 310 is required for full transcriptional activity of p65^28^. Our Western blot data showed that TNF-α increased acetylated p65 Lys 310 at low doses and decreased the acetylation of NF-κB at p65 Lys 310 at high doses, implied a complex role of TNF-α in human MSCs.

The reports that increased Wnt signaling may contribute to stem cell dysfunction in aged animals suggest that inhibiting Wnt/β-catenin signaling might improve stem cell function in the regenerative responses of aged tissues^36^,^37^. Wnt signaling has been reported to be positive, negative or stage dependent in the self-renewal and differentiation of MSCs^29^,^44^, and induces ageing and senescence in rat MSCs^57^,^58^. Both human MSCs and haematopoietic cells express Wnts (Fig. 3), it is reasonable to postulate that Wnts produced by one population contributes to supporting the properties of others in bone marrow niche. Our data showed that there is an age-related increase in β-catenin protein levels in human MSCs from old subjects and senescent MSCs (Fig. 7). Blocking β-catenin significantly decreased SA-β-Gal positive cells and increased ALP activity in MSCs (Fig. 7). These data imply that activating Wnt/β-catenin signaling might induce human MSC senescence and reduce osteoblast differentiation. Dependently cells and experimental conditions, it was reported that TNF-α stimulates nuclear β-catenin accumulation^53^ or promotes β-catenin degradation^52^ in human MSCs. Our result showed that TNF-α dose-dependently inclines β-catenin proteins in human marrow stromal KM101 cells, and NF-κB and β-catenin are two separate pathways (Fig. 6), together with the activating Wnt/β-catenin signaling might induce MSC senescence and reduce osteoblast differentiation, implying that TNF-α *via* NF-κB or β-catenin signaling performed as a negative factor in effects of haematopoietic cells on human MSCs.

In conclusion, our data demonstrate that there are paracrine interactions of haematopoietic cells, *via* soluble factors, such as TNF-α, PDGF-β or Wnts etc., on human MSCs; the age-related increase of TNF-α in haematopoietic cells suggests that immunosenescence, *via* the interactions of haematopoietic cells on mesenchymal stem cells, may be one of the extrinsic mechanisms of skeletal stem cell function decline during human skeletal ageing. Our data implied that besides the current approaches to intervene osteoporosis, such as targeting on osteoclasts to stop bone resorption or osteoblasts to increase bone formation, there may be a new approach that targets the interactions of haematopoietic cells on osteoblast precursors to identify potential intervention for osteoporosis and bone fracture, and to develop therapeutic strategies to prevent or restore skeletal tissue degeneration and loss in the ageing population.

## Methods

### Human bone marrow mononuclear (MNCs) cells

Bone marrow samples were obtained from discarded femoral tissues during primary arthroplasty for osteoarthritis according to an established Institutional Review Board (IRB) protocol approved by Partners Human Research Committee (PHRC) of Partners Healthcare, Boston, MA. All identifying information is destroyed once we obtain the discarded tissue; therefore, our study was found to be exempt from the need for written informed consent by PHRC. As in our previous studies^45^,^59^, exclusion criteria included infectious diseases and any known disorder that may affect bone such as: hyperthyroidism, osteomyelitis, primary hyperparathyroidism, Paget’s disease of bone, rheumatoid arthritis, cancer, and use of medications that affect bone, such as glucocorticoids, bisphosphonates, and non-steroidal anti-inflammatory drugs. Low-density human bone marrow mononuclear (MNCs) cells were isolated by density centrifugation with Ficoll/Histopaque 1077 from human bone marrows. Not all specimens could be included in every experiment, due to the surgical schedule and numbers of cells needed for each assay. In each experiment, standardized conditions were used for all samples, *e.g.* early cell passage, identical medium, serum, and regents. We used Magnetic activated cell sorting (MACS) separation for collection of mature haematopoietic cells and their committed precursors, such as T cells, B cells, NK cells, dendritic cells, monocytes, granulocytes, erythroid cells, from human bone marrow MNCs. Cells were first labeled with a cocktail of biotin-conjugated antibodies targeting a panel of so-called “lineage” antigens: CD2, CD3, CD11b, CD14, CD15, CD16, CD19, CD56, CD123, and CD235a (glycophorin A), and then labeled with Anti-Biotin MicroBeads and separated with magnetic separation columns (Miltenyi Biotec, CA). Our results showed that 93-98% of MNCs are lineage^+^ haematopoietic cells and CFU-Fs were enriched (33-fold) in Lin^-^ cells compared with Lin^+^ cells of MNCs obtained from a 64-year-old male (64M) subject.

### Preparation of human mesenchymal stem cells (MSCs)

Adherent human MSCs were prepared from MNCs as we previous described^22^,^44^. Human MSCs were expanded in monolayer culture with phenol red-free α-MEM medium, 10% heat-inactivated fetal bovine serum (FBS-HI) and antibiotics (100 U/ml penicillin, and 100 μg/ml streptomycin) (Gibco BRL, Invitrogen, Carlsbad, CA). Human MSCs from 5 samples (17 M, 44 F, 72 F, 72 M, 76 F) were analyzed for expression of CD166, CD90, CD105, STRO-1, CD34, and CD45 with flow cytometry. The cells were labeled with FITC and PE -conjugated antibodies against the cell surface markers, and 2-color analysis was performed. All the samples expressed CD166, CD90, CD105, and STRO-1. The level of expression of CD34 and CD45 (markers of hematopoietic lineage) was very dim in all samples, and in some cases negative when compared to controls. There was no significant difference between the percent of cells or level of expression of CD166, CD90, CD105, and STRO-1 for all the MSCs samples.

### Co-cultures of haematopoietic-mesenchymal stem cells

The interaction of haematopoietic-stromal cells was tested in a co-culture system as shown in Supplementary Figure 1b. Human MNCs were put inside of the cell culture inserts (polycarbonate membrane with 0.4 μm pore size, Nalge Nunc International, NY) and human MSCs were cultured on the bottom of cell culture dish. The 0.4 μm pore size of cell culture insert allows proteins or small molecules to transport through the polycarbonate membrane, but not cells.

### Cell proliferation and senescence assays

For MSCs proliferation, proliferation rate of hMSCs obtained from each subject were measured by plating second passage cells 1 × 10^4^ cells/35 mm dishes in triplicate co-cultured with MNCs or treated with TNF-α. Cell number was counted with a hemocytometer at day 7. For senescent marker senescence-associated β-galactosidase, hMSCs obtained from each subject were plated at 2 × 10^4^ cells/35 mm dish (n = 4). After 7 days co-cultured with MNCs or treated with TNF-α, MSCs were stained for SA-β-galactosidase as we described^22^, and data were presented as % of SA-β-Gal^+^ cells in total cells.

### Analyses of osteoblast differentiation

MSCs were cultured in MEM-α with 1% FBS-HI and antibiotics (100 U/ml penicillin and 100 μg/ml streptomycin, Invitrogen) plus 10 nM dexamethasone, 5 mM β-glycerophosphate, and 50 μg/mL ascorbate-2-phosphate (Sigma). Cultures were harvested for biochemical alkaline phosphatase activity assays and RNA after one week culture in osteogenic medium as we described^59^.

### RNA isolation and semi-quantitative RT-PCR

Total RNA was isolated from human MSCs or MNCs with Trizol reagent (Invitrogen). For semi-quantitative RT-PCR, 2 μg of total RNA was reverse-transcribed into cDNA with M-MLV reverse transcriptase (Promega), following the manufacturer’s instructions. Concentration of cDNA and amplification conditions were optimize to reflect the exponential phase of amplification. In general, one-twentieth of the cDNA was used in each 50 μL PCR reaction (30-40 cycles of 94 °C for 1 minute, 55-60 °C for 1 minute, and 72 °C for 2 minutes) as we described^22^. The gene-specific primers (Supplementary Table 1) were used for amplification with Promega GoTaq Flexi DNA Polymerase. PCR products were quantified by densitometry of captured gel images with Gel Logic 200 Imaging System and measured by KODAK Molecular Imageing Software, following the manufacturer’s instructions (Carestream Health, Rochester, NY). Quantitative data were expressed by normalizing the densitometric units to *GAPDH* (internal control).

### Western blot analysis

The whole-cell lysates were prepared with lysis buffer containing 150 mM NaCl, 3 mM NaHCO_3_, 0.1% of Triton X-100 and a mixture of protease inhibitors (Roche Diagnostics, CA) plus 2 mM Na_4_VO_4_ and 5 mM NaF. The whole-cell lysates were homogenized with a Kontes’ Pellet Pestle, frozen in −80 °C for minimum 25 minutes and then separated from insoluble cell materials by centrifugation at 16,000 g in a bench-top Eppendorf centrifuge at 4 °C. Nuclear fractions were obtained as we described^29^. Protein concentration was determined with the BCA system (Pierce, Rockford, IL). The Western blotting was performed as we previously described^29^,^44^,^60^. The primary antibody anti-β-catenin (E5) and anti-β-actin were purchased from Santa Cruz Biotechnology (Dallas, TX). NF-κB signaling was analyzed with NF-κB p65 antibody sampler kit (Cell Signaling, Danvers, MA). The second antibody anti-mouse IgG-HRP was purchased from Santa Cruz Biotechnology. The antibody-associated protein bands were revealed with Western blotting luminol reagent (Santa Cruz Biotechnology, Dallas, TX).

### Transient transfection of siRNA

Transient transfection of β-catenin siRNA, RelA/p65 siRNA (Stealth RNAi duplex siRNA, Invitrogen) or control siRNA (SiRNA-A, Santa Cruz Biothech., Inc.) into human MSCs or KM101 cells was performed by electroporation with the Human MSC Nucleofector Kit (Lonza) according to the manufacturer’s instruction and as we described^44^. In briefly, cells were harvested by trypsinization, and resuspended one million cells in 100 μL of human MSC nucleofector solution with 100 pmole of β-catenin, p65 or control siRNA. Electroporation was performed in Nucleofector^TM^ II with program U-23 provided by Lonza/Amaxa Biosystems. Immediately after electroporation, MSCs were transferred into 6 or 24-well plates or 60 mm dishes in MEM-α with 10% FBS-HI and KM101 cells were transferred into 60 mm dishes in IMEM with 10% FBS-HI. After confluence, cells were used for Western blot assays (60 mm dishes) and SA-β-Gal staining (6-well plates) or the medium was changed into osteogenic medium for 7 days to analyze osteogenic marker ALP enzyme activity (24-well plates).

### Statistical analyses

The experiments were performed three or more times independently. Data are presented as mean values ± SEM of all experiments or a representative result of three or more experiments. Quantitative data were analyzed by GraphPad InStat software with one-way ANOVA, Student’s two-tail unpaired t-test, Mann-Whitney test or Pearson correlation test. A value of p < 0.05 was considered significant.

## Additional Information

**How to cite this article**: Zhou, S. Paracrine effects of haematopoietic cells on human mesenchymal stem cells. *Sci. Rep.* **5**, 10573; doi: 10.1038/srep10573 (2015).

## Supplementary Material

Supporting Information
