# Supplementary material for: Paracrine effects of haematopoietic cells on human mesenchymal stem cells
Source: Sci Rep. 2015 Jun 1;5:10573. doi: 10.1038/srep10573 (PMC4450757; doi:10.1038/srep10573)
Supplement: Supporting Information [file srep10573-s1.pdf]

Paracrine effects of haematopoietic cells on human mesenchymal stem cells

Shuanhu Zhou

Supplementary Information

Supplementary Fig. 1

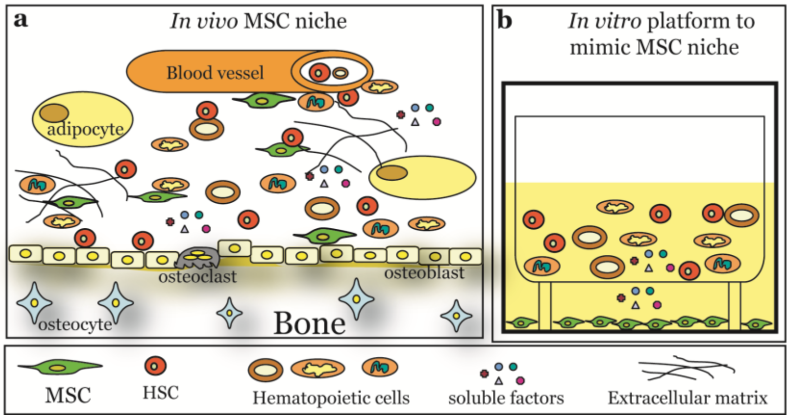

Supplementary Figure 1. The niche of human MSCs. (**a**) Bone and blood formation are intertwined in bone marrow, therefore haematopoietic cells and bone cells could be extrinsic factors/niche for each other. (**b**) An *in vitro* transwell co-culture model of haematopoietic cells and MSCs as a platform to mimic the paracrince effects of haematopoietic cells on hMSCs *in vivo*. Human haematopoietic cells were placed in cell culture inserts, and MSCs were cultured on the bottom of the dishes. The 0.4 μm pore size of cell culture insert allows proteins, but not cells, to transport through the polycarbonate membrane.

Supplementary Fig. 2

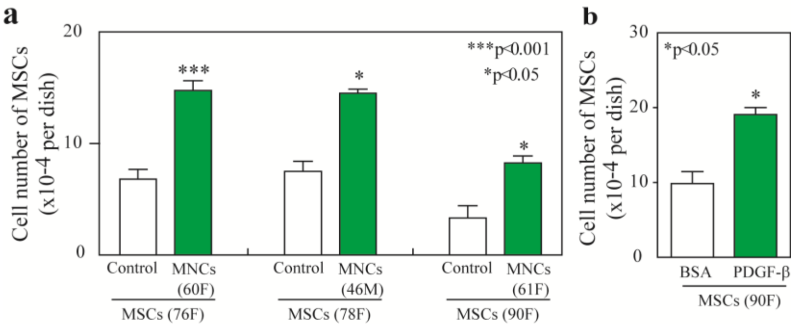

Supplementary Figure 2. Haematopoietic cells stimulate proliferation of human MSCs. Human MSCs were seeded in 1x10⁴ cells/35 mm dish at day 0 and cell number was accounted at day 7. **(a)** Human MSCs (76F) ± MNCs (60F) (empty controls, n=6, MNCs inserts, n=6, p<0.001, t-test); hMSCs (78F) ± MNCs (46M) (empty controls, n=3, MNCs inserts, n=4, p<0.05, t-test); hMSCs (90F) ± MNCs (61F) (empty controls, n=4, MNCs inserts, n=3, p<0.05, t-test). (**b**) After 7 days treatment, 10 ng/mL of PDGF-β stimulated proliferation of hMSCs (90F) (*p<0.05, n=3, Mann-Whitney test).

Supplementary Fig. 3

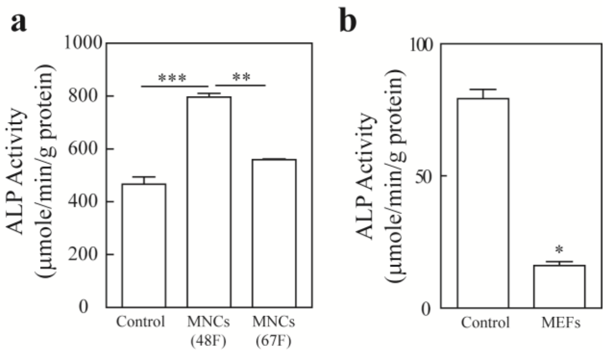

Supplementary Figure 3. Haematopoietic cells, but not MEFs, stimulate osteoblast differentiation of human MSCs. (**a**) After 7 days in osteogenic medium, ALP activity of MSCs (76M) ± MNCs; MNCs inserts (48F, n=3) *vs*. empty insert controls (n=11) (***p<0.001) or MNCs inserts (67F, n=3) (**p<0.01) (ANOVA); there was no significant different between MNCs inserts (67F) and empty insert controls. (**b**) Effects of MEFs on ALP activity of MSCs (90F); MEFs inserts (n=3) *vs*. empty insert controls (n=6) (*p<0.05, Mann-Whitney Test).

Supplementary Fig. 4

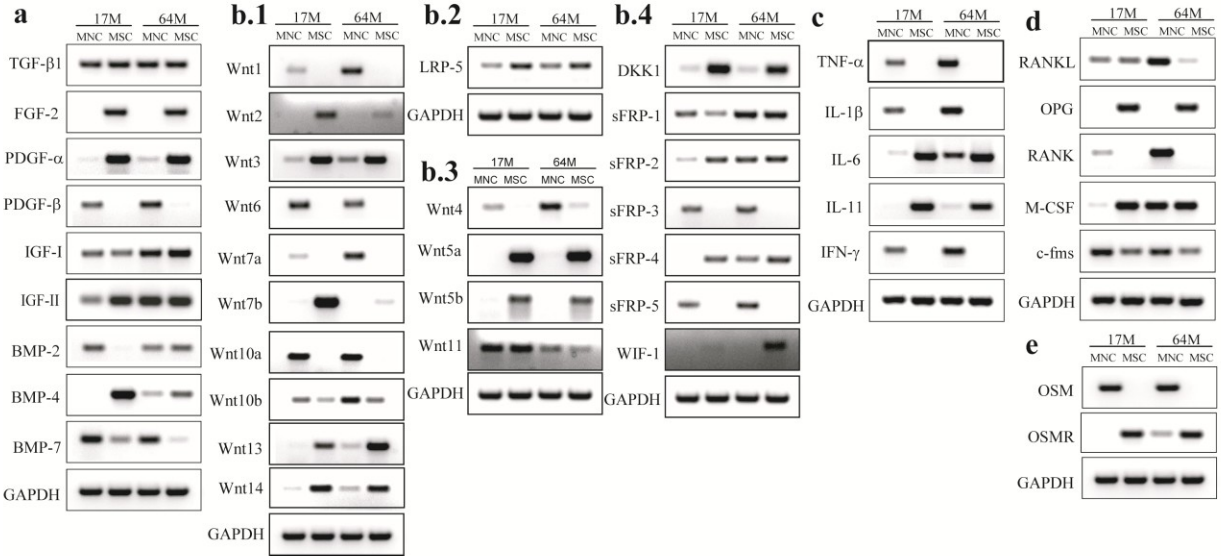

Supplementary Figure 4. The gene profile of human MNCs and MSCs. RT-PCR was used to evaluate the gene profile in human MSCs and MNCs obtained from two subjects, a young male (17M) and an old male (64M) subjects. The gene-specific primers (Supplementary Table 1) were used for amplification with Promega GoTaq Flexi DNA Polymerase.

Supplementary Fig. 5

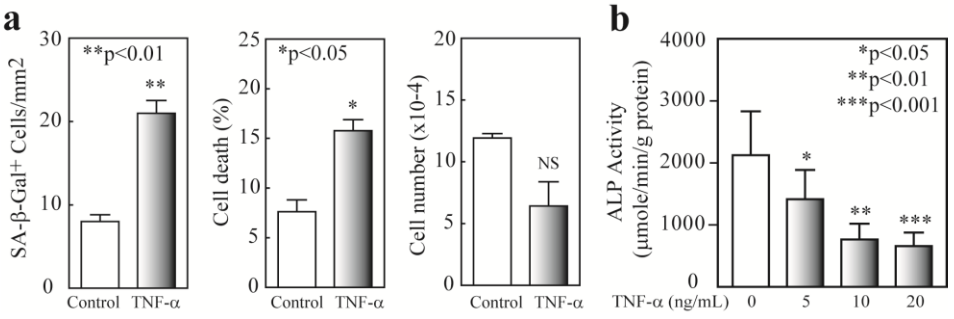

Supplementary Figure 5. The effects of TNF-α on human MSCs. (**a**) Under the same treatment conditions as the described in Figure 5, the effects of TNF-α on SA-β-Gal^{+} cells (**p<0.01, TNF-α, n=3 *vs*. control, n=4, t-test), cell death (*p<0.05, TNF-α, n=3 *vs*. control, n=3, t-test), and proliferation (no significant different between TNF-α treatment and control,

n=3) in hMSCs obtained from a 76-year-old male subject. (**b**) TNF-α significantly inhibited ALP activity of hMSCs (47F) in a dose-dependent manner (TNF-α treatments *vs*. control, n=3, ANOVA).

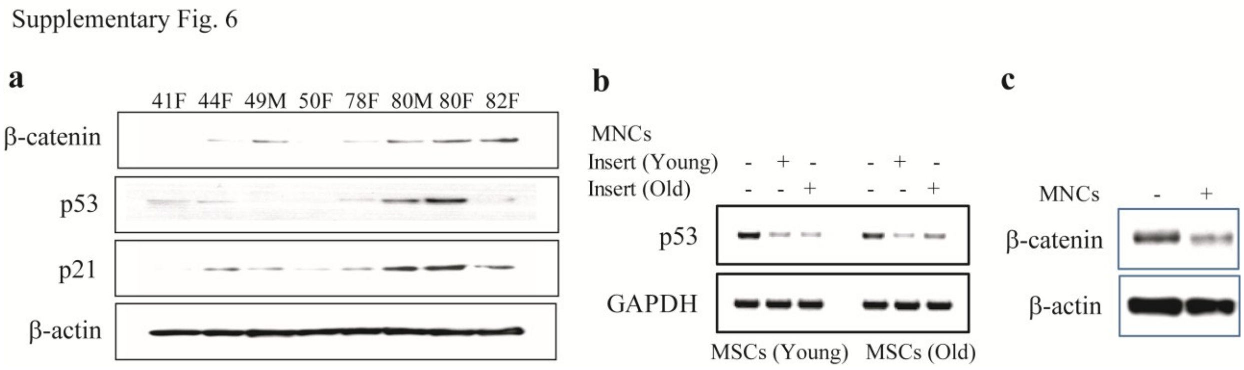

Supplementary Figure 6. Effects of age and MNCs on β-catenin, p53 and p21 in hMSCs. (**a**) Western blot showed that the age-related increases in constitutive β-catenin protein levels as well as p53 and p21 protein levels in hMSCs (41-82-year-old subjects). Co-cultures of hMSCs with MNCs showed that (**b**) MNCs (young, 37M; old, 64M) co-culture inserts down-regulated p53 gene expression in hMSCs (young, 37M; old, 64M) after 7 days co-culture and (**c**) MNCs (54M) down-regulated β-catenin protein in hMSCs (21F) after 24 hours co-culture.

Supplementary Fig. 7

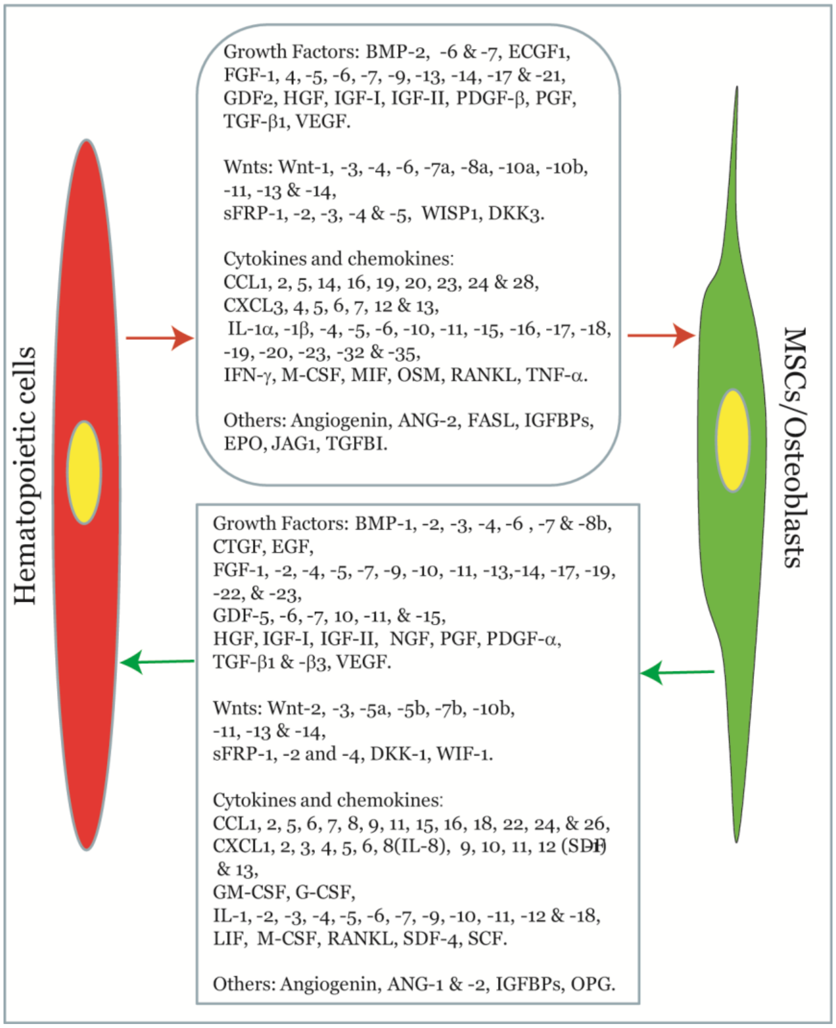

Supplementary Figure 7. Summary of the paracrine interactions between haematopoietic cells and mesenchymal stem cells. Secreted factors that may involve the paracrine interactions between haematopoietic cells and MSCs and/or osteoblasts are summarized from our study and literature as listed.

Abbreviations: ANG – angiopoietin, BMP- bone morphogenetic proteins, CCL - chemokine (C-C motif) ligand, CTGF - connective tissue growth factor, CXCL - chemokine (C-X-C motif) ligand, DKK – Dickkopf, ECGF - endothelial cell growth factor, EGF - epidermal growth factor,

EPO – erythropoietin, FASL - Fas ligand, FGF - fibroblast growth factors, GDF - growth differentiation factor, GM-CSF - granulocyte-macrophage colony-stimulating factor, G-CSF - granulocyte-colony stimulating factor, HGF - hepatocyte growth factor, IGF - insulin-like growth factor, IGFBP - insulin-like growth factor-binding protein, LIF - leukemia inhibitory factor, IL – Interleukin, INF – Interferon, JAG – jagged, M-CSF - macrophage colony-stimulating factor, MIF - macrophage migration inhibitory factor, NGF - nerve growth factor, OPG – osteoprotegerin, OSM - oncostatin M, PGF - placental growth factor, PDGF - platelet-derived growth factor, RANKL - receptor activator of nuclear factor kappa B ligand, RANK - receptor activator of nuclear factor kappa B, SCF - Stem Cell Factor, SDF-1 - stromal cell-derived factor 1, TGF - transforming growth factor, TGFBI - transforming growth factor, beta-induced, TNF - tumor necrosis factor, VEGF - vascular endothelial growth factor, sFRP - secreted frizzled-related protein, WIP-1 - Wnt inhibitory factor-1, WISP1 - Wnt-1 induced secreted protein 1.

References:

Bronckaers, A., *et al.* Mesenchymal stem/stromal cells as a pharmacological and therapeutic approach to accelerate angiogenesis. Pharmacol. Ther. **143**, 181-196 (2014).

Korf-Klingebiel, M., *et al.* Bone marrow cells are a rich source of growth factors and cytokines: implications for cell therapy trials after myocardial infarction. Eur. Heart J. **29**, 2851-2858 (2008).

Kupcova Skalnikova H. Proteomic techniques for characterisation of mesenchymal stem cell secretome. Biochimie. **95**, 2196-2211 (2013).

Lorenzo, J., Horowitz, M., Choi, Y. Osteoimmunology: interactions of the bone and immune system. Endocr. Rev. **29**, 403-440 (2008).

Nakashima, T., & Takayanagi, H. Osteoimmunology: crosstalk between the immune and bone systems. J. Clin. Immunol. **29**, 555-567 (2009).

Overman, J. R. *et al.* Growth factor gene expression profiles of bone morphogenetic protein-2-treated human adipose stem cells seeded on calcium phosphate scaffolds *in vitro*. Biochimie. **95**, 2304-2313 (2013).

Pacifici, R. Osteoimmunology and its implications for transplantation. Am. J. Transplant. **13**, 2245-2254 (2013).

Shiozawa, Y., & Taichman, R. S. Getting blood from bone: an emerging understanding of the role that osteoblasts play in regulating hematopoietic stem cells within their niche. Exp. Hematol. **40**, 685-694 (2012).

Taichman, R. S. Blood and bone: two tissues whose fates are intertwined to create the hematopoietic stem-cell niche. Blood. **105**, 2631-2639 (2005);

Supplementary Table 1. PCR primers

Gene	Primer sequence (5'→3')	Size (bp)	Ref.
ALP	F: GCGAACGTATTTCTCCAGACCCAG<br>R: TTCCAAACAGGAGAGTCGCTTCA	367	Winn *et al.*, 1999
BMP-2	F: CATCCCAGCCCTCTGAC<br>R: CTTTCCCACCTGCTTGCA	493	Moutsatsos et al., 2001
BMP-4	F: GACCTATGGAGCCATTCCGTA<br>R: TCAGGGATGCTGCTGAGGTT	585	M22490*
BMP-7	F: 5-TTCCCCTCCCTATCCCCAACTTT-3<br>R: 5-TTTTCCTTTCGCACAGACACC-3	313	Chubinskaya, *et al.*, 2000
BSP	F: TCAGCATTTTGGGAATGGCC<br>R: GAGGTTGTTGTCTTCGAGGT	657	D'Ippolito *et al.*, 2006
c-fms	F: CAGATTGGTATAGTCCCGCTCTCT<br>R: TCCAACTACATTGTCAAGGGCAAT	360	Kirma *et al.*, 2007
COL I	F: GGGTGACCGTGGTGAGA<br>R: CCAGGAGAGCCAGAGGTCC	194	Büttner *et al.*, 2004
DKK1	F: TGGAATATGTGTGTCTTCTG<br>R: AACCTTCTTGTCCTTTGGTG	155	Mueller *et al.*, 2005
FGF-2	F: GGCTTCTTCCTGCGCATCCA<br>R: GCTCTTAGCAGACATTGGAAGA	352	Baguma-Nibasheka *et al.*, 2007
IGF-I	F: TGGGCTTGCGGCCCACGTAGTA<br>R: AGAGCCTGCGCAATGGAATAAA	533	Gordeladze *et al.*, 2002
IGF-II	F: CTGTGCTACCCCCGCCAAGT<br>R: ACGTTTGGCCTCCCTGAACG	214	Sayer *et al.*, 2005
IL-1β	F: CATGGACAAGCTGAGGAAGA<br>R: TTCAACACGCAGGACAGGTA	370	Hennige *et al.*, 2008
IL-6	ATGAACTCCTTCTCCACAAGCGC<br>GAAGAGCCCTCAGGCTGGACTG	627	Stark *et al.*, 1993
IL-11	F: ACTGCTGCTGCTGAAGACTCGGCTGTGA<br>R: ATGGGGAAGAGCCAGGGCAGAAGTCTGT	321	Suen *et al.*, 1994
IFN-γ	F: GCGAAAAAGGAGTCAGATGC<br>R: CAAACCGGCAGTAACTGGAT	417	NM_000619.2#
GAPDH	F: ACCACAGTCCATGCCATCAC<br>R: TCCACCACCCTGTTGCTGTA	451	Pattyn et al., 2003
LRP-5	F: CTTCCAGTTTTCCAAGGGA<br>R: AGTCCACAATGATCTTCCGGGT	366	Zhou *et al.*, 2004
M-CSF	F:ATGACAGACAGGTGGAACTGCCAGTGTAGAGG<br>R: TCACACAACTTCAGTAGGTTCAGGTGATGGGC	495	Clontech
OPG	F: GAACCCCAGAGCGAAATACA<br>R: CGCTGTTTTCACAGAGGTCA	441	Makhluf *et al.*, 2000
OSM	F: CGTATCCAAGGCCTGGATGTT<br>R: GCCCTCCAGCTTGCGCTGAAA	393	Huang *et al.*, 2009
OSMR	F:GGAGGAAGTCAGTGTACAAGA<br>R: TACAGTGCAAAGTCTTGAAGTC	362	Götherström *et al.*, 2010
PDGFα	F: CTGGAGATAGACTCCGT<br>R: CCTGACGTATTCCACCT	335	Yoshida *et al.*, 1992

PDGFβ	F: CCCGGAGTCGGCATGAA<br>R: TTTCTCACCTGGACAGGTCG	475	Yoshida *et al.*, 1992
RANK	F: CCTACGCACAAGGCGAAGATGC<br>R: CGTAGACCACGATGATGTCGCC	702	Atkins *et al.*, 2000
RANKL	F: ATCCCATCTGGTTCCCATAA<br>R: CCCTGACCAATACTTGGTGC	276	Eslami *et al.*, 2011
RUNX2	F: GTTTGTTCTCTGACCGCCTC<br>R: CCAGTTCTGAGGCACCTGAAA	317	D'Ippolito *et al.*, 2006
sFRP-1	F: TACGTACCTGGTGGACATGC<br>R: AAGGACGTGCCGATAAACAG	497	Yates, 2004
sFRP-2	F: CTAGCGCCGCTCTTCGTGTACCTG<br>R: CAGCGTCTTGCCCGACCAGATCCA	386	Yates, 2004
sFRP-3	F: GGATCGGTGTTTTCAGCATT<br>R: CCGTGGTAGCTGCTCACTTT	501	Yates, 2004
sFRP-4	F: AACTTTCACACCGCTCATCC<br>R: GATATCCTTTCCCGGCCTAC	602	Yates, 2004
sFRP-5	F: TTCATGTGCCTGGTGGTGGGC<br>R: TACACGTGCGACAGGGACACC	235	Yates, 2004
TERT	F: AGCCAGTCTCACCTTCAACCGC<br>R: GGAGTAGCAGAGGGAGGCCG	272	D'Ippolito *et al.*, 2006
TGF-β1	F: CAGAAATACAGCAACAATTCCTGG<br>R: TTGCAGTGTGTTATCCGTGCTGTC	186	Nagineni *et al.*, 2002
TNF-α	F: GCGTGGAGCTGAGAGATAAC<br>R: GATGTTCGTCCTCCTCACAG	360	Hennige *et al.* 2008
TNFRI	F: ACCAAGTGCCACAAAGGAAC<br>R: CTGCAATTGAAGCACTGGAA	263	Sawanobori *et al.*, 2003
TNFRII	F: TTCGCTCTTCCAGTTGGACT<br>R: CACCAGGGGAAGAATCTGAG	399	Sawanobori *et al.*, 2003
WIF-1	F: CCGAAATGGAGGCTTTTGTA<br>R: GTGTCTTCCATGCCAACCTT	451	Lin *et al.*, 2006
Wnt1	F: TGCACGCACACGCGCGTACTGCAC<br>R: CAGGATGGCAAGAGGGTTCATG	246	Katoh, 2003
Wnt2	F: AAAGGAAAGGATGCCAGAGC<br>R: CCCACAGCACATGACTTCAC	398	Yates, 2004
Wnt3	F: GSCCACATGCACCTCAAATG<br>R: GATGCAGTGGCATTTTTCCT	401	Yates, 2004
Wnt4	F: ACCTGGAAGTCATGGACTCG<br>R: GCCTCATTGTTGTGGAGGTT		Yates, 2004
Wnt5a	F: CCAACTGGCAGGACTTTCTC<br>R: GCAAAGCGGTAGCCATAGTC	368	Yates, 2004
Wnt5b	F: AGATCGTGGACCAGTACATCTG<br>R: TTACGGAACCCATCTACATTCTG	504	Saitoh and Katoh, 2002
Wnt6	F: AGAAGCTGCCTCCATTTCG<br>R: GTCACAGGCAGAGGCTGAG	386	Yates, 2004
Wnt7a	F: GAGAAGCAAGGCCAGTACCA<br>R: TAGTTGGGCGACTTCTCGAT	424	Yates, 2004
Wnt7b	F: GAGCCAACATCATCTGCAAC<br>R: GGAGAAGTCGATGCCGTAAC	391	Yates, 2004
Wnt10a	F: CCCAATGACATTCTGGACCT<br>R: TAAGCGGTGCAGCTTCCTAC	410	Yates, 2004

Wnt10b	F: GAATGCGAATCCACAACAACAG<br>R: TTGCGGTTGTGGGTATCAATGAA	195	Yates, 2004
Wnt11	F: CGTGTGCTATGGCATCAAGT<br>R: CGCATCAGTTTATTGGCTTG	509	Yates, 2004
Wnt13	F: AAGATGGTGCCAACTTCACCG<br>R: CTGCCTTCTTGGGGGCTTTGC	320	Yates, 2004
Wnt14	F: GGGTGTGAAGGTGATCAAGG<br>R: CACCCGGCTCTGTGTGTTAT	396	Yates, 2004

Note: F = Forward, R = Reverse. * Designed with Primer3 program. #Designed with Primer-BLAST.

Supplementary Reference:

Atkins, G. J., Haynes, D. R., Graves, S. E., *et al.* Expression of osteoclast differentiation signals by stromal elements of giant cell tumors. *J. Bone Miner. Res.* **15**, 640-649 (2000).

Baguma-Nibasheka, M., Barclay, C., Li, A. W., Geldenhuys, L., Porter, G. A., Blay, J., Casson, A. G., Murphy, P. R. Selective cyclooxygenase-2 inhibition suppresses basic fibroblast growth factor expression in human esophageal adenocarcinoma. *Mol. Carcinog.* **46**, 971-980 (2007).

Büttner, C., Skupin, A., Rieber, E. P. Transcriptional activation of the type I collagen genes COL1A1 and COL1A2 in fibroblasts by interleukin-4: analysis of the functional collagen promoter sequences. *J. Cell. Physiol.* **198**, 248-258 (2004).

Chubinskaya, S., Merrihew, C., Cs-Szabo, G., Mollenhauer, J., McCartney, J., Rueger, D. C., Kuettner, K. E. Human articular chondrocytes express osteogenic protein-1. *J. Histochem. Cytochem.* **48**, 239-250 (2000).

D'Ippolito, G., Diabira, S., Howard, G. A., Roos, B. A., Schiller, P. C. Low oxygen tension inhibits osteogenic differentiation and enhances stemness of human MIAMI cells. *Bone* **39**, 513-522 (2006).

Eslami, B., Zhou, S., Van Eekeren, I., *et al*. Reduced osteoclastogenesis and RANKL expression in marrow from women taking alendronate. *Calcified Tissue Intl*. **88**, 272-280 (2011).

Gordeladze, J. O., Drevon, C. A., Syversen, U., Reseland, J. E. Leptin stimulates human osteoblastic cell proliferation, de novo collagen synthesis, and mineralization: Impact on differentiation markers, apoptosis, and osteoclastic signaling**.** J. Cellular Biochem. **85**, 825-836 (2002).

Götherström, C., Chan, J., O'Donoghue, K., Fisk, N. M. Identification of candidate surface antigens for non-invasive prenatal diagnosis by comparative global gene expression on human fetal mesenchymal stem cells. *Mol. Hum. Reprod.* 16, 472-480 (2010).

Hennige, A. M., Staiger, H., Wicke, C., Machicao, F., Fritsche, A., Häring, H. U., Stefan, N. Fetuin-A induces cytokine expression and suppresses adiponectin production. *PLoS One* **3**, e1765 (2008).

Huang, F. M., Tsai, C. H., Yang, S. F., Chang, Y. C. The upregulation of oncostatin M in inflamed human dental pulps. *Int. Endod. J.* **42**, 627-631 (2009).

Katoh, M. Expression and regulation of WNT1 in human cancer: up-regulation of WNT1 by β-estradiol in MCF-7 cells. *Int. J. Oncol.* **22**, 209-212 (2003).

Kirma, N., Hammes, L. S., Liu, Y. G., Nair, H. B., Valente, P. T., Kumar, S., Flowers, L. C., Tekmal, R. R. Elevated expression of the oncogene c-fms and its ligand, the macrophage colony-stimulating factor-1, in cervical cancer and the role of transforming growth factor-beta1 in inducing c-fms expression. *Cancer Res.* **67**, 1918-1926 (2007).

Lin, Y.C., You, L., Xu, Z., He, B., Mikami, I., Thung, E., Chou, J., Kuchenbecker, K., Kim, J., Raz, D., Yang, C. T., Chen, J. K., Jablons, D. M. Wnt signaling activation and WIF-1 silencing in nasopharyngeal cancer cell lines. *Biochem. Biophys. Res. Commun.* **341**, 635-40 (2006).

Makhluf, H. A., Mueller, S. M., Mizuno, S., Glowacki, J. Age-related decline in osteoprotegerin expression by human bone marrow cells cultured in three-dimensional collagen sponges. *Biochem. Biophys. Res. Commun.* **268**, 669-672 (2000).

Moutsatsos, I. K., Turgeman, G., Zhou, S., Kurkalli, B. G., Pelled, G., Tzur, L., Kelley, P., Stumm, N., Mi, S., Müller, R., Zilberman, Y., Gazit, D. Exogenously regulated stem cell-mediated gene therapy for bone regeneration. Mol Ther. **3**, 449-461 (2001).

Mueller, W., Lass, U., Wellmann, S., Kunitz, F., von Deimling, A. Mutation analysis of DKK1 and in vivo evidence of predominant p53-independent DKK1 function in gliomas. *Acta Neuropathol.* **109**, 314-320 (2005).

Nagineni, C. N., Detrick, B., Hooks, J. J. Transforming growth factor-β expression in human retinal pigment epithelial cells is enhanced by Toxoplasma gondii: a possible role in the immunopathogenesis of retinochoroiditis. *Clin. Exp. Immunol.* **128**, 372-378 (2002).

Pattyn, F., Speleman, F., De Paepe, A., Vandesompele, J. RTPrimerDB: the real-time PCR primer and probe database. *Nucleic Acids Res.* **31**, 122-123 (2003).

Sawanobori, M., Yamaguchi, S., Hasegawa, M., Inoue, M., Suzuki, K., Kamiyama, R., Hirokawa, K., Kitagawa, M. Expression of TNF receptors and related signaling molecules in the bone marrow from patients with myelodysplastic syndromes. *Leuk. Res.* **27**, 583-591 (2003).

Sayer, R. A., Lancaster, J. M., Pittman, J., Gray, J., Whitaker, R., Marks, J. R., Berchuck, A. High insulin-like growth factor-2 (IGF-2) gene expression is an independent predictor of poor survival for patients with advanced stage serous epithelial ovarian cancer. *Gynecol.*

Oncol. **96**, 355-361 (2005).

Saitoh, T., Katoh, M. Expression and regulation of WNT5A and WNT5B in human cancer: up-regulation of WNT5A by TNFalpha in MKN45 cells and up-regulation of WNT5B by beta-estradiol in MCF-7 cells. *Int. J. Mol. Med.* **10**, 345-349 (2002).

Stark, R., Andre, C., Thierry, D., Cherel, M., Galibert, F., Gluckman, E. The expression of cytokine and cytokine receptor genes in long-term bone marrow culture in congenital and acquired bone marrow hypoplasias. *Br. J. Haematol.* **83**, 560-566 (1993).

Suen, Y., Chang, M., Lee, S. M., Buzby, J. S., Cairo, M. S. Regulation of interleukin-11 protein and mRNA expression in neonatal and adult fibroblasts and endothelial cells. *Blood* **84**, 4125-4134 (1994).

Winn, S. R., Randolph, G., Uludag, H., Wong, S. C., Hair, G. A., Hollinger, J. O. Establishing an immortalized human osteoprecursor cell line: OPC1. *J. Bone Miner. Res.* **14**, 1721-1733 (1999).

Yates, K. E. Demineralized bone alters expression of Wnt network components during chondroinduction of post-natal fibroblasts. *Osteoarthritis Cartilage* **12**, 497-505 (2004).

Yoshida, M., Tanihara, H., Yoshimura, N. Platelet-derived growth factor gene expression in cultured human retinal pigment epithelial cells. *Biochem. Biophys. Res. Commun.* **189**, 66-71 (1992).

Zhou, S., Eid, K., Glowacki, J. Cooperation between TGF-β and Wnt pathways during chondrocyte and adipocyte differentiation of human marrow stromal cells. *J. Bone Miner. Res.* **19**, 463-470 (2004).
